# hESC Expansion and Stemness Are Independent of Connexin Forty-Three-Mediated Intercellular Communication between hESCs and hASC Feeder Cells

**DOI:** 10.1371/journal.pone.0069175

**Published:** 2013-07-26

**Authors:** Jin-Su Kim, Daekee Kwon, Seung-Taeh Hwang, Dong Ryul Lee, Sung Han Shim, Hee-Chun Kim, Hansoo Park, Won Kim, Myung-Kwan Han, Soo-Hong Lee

**Affiliations:** 1 Department of Biomedical Science, CHA University, Sungnam, Republic of Korea; 2 Department of Biomedical Science, CHA University, Seoul, Republic of Korea; 3 Department of Orthopaedics, Bundang CHA Hospital, Sungnam, Republic of Korea; 4 Department of Integrative Engineering, Chung-Ang University, Seoul, Republic of Korea; 5 Department of Internal Medicine, Chonbuk National University, Jeonju, Republic of Korea; 6 Departments of Microbiology and biochemistry, Chonbuk National University, Jeonju, Republic of Korea; University of California, San Diego, United States of America

## Abstract

**Background:**

Human embryonic stem cells (hESCs) are a promising and powerful source of cells for applications in regenerative medicine, tissue engineering, cell-based therapies, and drug discovery. Many researchers have employed conventional culture techniques using feeder cells to expand hESCs in significant numbers, although feeder-free culture techniques have recently been developed. In regard to stem cell expansion, gap junctional intercellular communication (GJIC) is thought to play an important role in hESC survival and differentiation. Indeed, it has been reported that hESC-hESC communication through connexin 43 (Cx43, one of the major gap junctional proteins) is crucial for the maintenance of hESC stemness during expansion. However, the role of GJIC between hESCs and feeder cells is unclear and has not yet been reported.

**Methodology/Principal Findings:**

This study therefore examined whether a direct Cx43-mediated interaction between hESCs and human adipose-derived stem cells (hASCs) influences the maintenance of hESC stemness. Over 10 passages, hESCs cultured on a layer of Cx43-downregulated hASC feeder cells showed normal morphology, proliferation (colony growth), and stemness, as assessed by alkaline phosphatase (AP), *OCT4* (*POU5F1*-Human gene Nomenclature Database), *SOX2*, and *NANOG* expression.

**Conclusions/Significance:**

These results demonstrate that Cx43-mediated GJIC between hESCs and hASC feeder cells is not an important factor for the conservation of hESC stemness and expansion.

## Introduction

Human embryonic stem cells (hESCs) are pluripotent stem cells derived from the inner cell mass (ICM) of human blastocysts [1], [2]. These cells have pluripotency and the ability to self-renew *in vitro*. Under certain conditions, hESCs are able to differentiate into all derivatives of the three primary germ layers [3], [4]. These include more than 220 cell types found in the adult body [5]. Therefore, hESCs have attracted considerable attention in many fields of research, such as basic stem cell biology, *in vitro* drug screening, patient-specific cell therapies, and so forth [6].

Ever since Thomson's group first achieved success in hESC culture in 1998, a mouse feeder cell layer has commonly been employed for hESC expansion [1]. To avoid the contamination issues presumably induced by feeder cells, feeder-free culture and suspension culture techniques have been suggested in recent years [7], [8], [9]. For example, feeder-free cultures without the support of feeder cells have been established through the dual utilisation of mouse embryonic fibroblast (MEF) conditioned medium and matrigel coatings [7]. Moreover, a commercially available product composed of mTeSR™ medium and specific extracellular matrix (ECM) components is now available for the feeder-free culture of hESCs [10]. However, these feeder-free culture techniques can potentially cause genetic aberrations in hESCs by increasing their chromosomal instability and susceptibility to mitochondrial diseases. Therefore, it is very difficult to produce large numbers of clinical grade hESCs through the use of such feeder-free techniques [11], [12].

On the other hand, suspension culture techniques have the advantage of allowing mass production of hESCs [13]. Nonetheless, suspension culture is associated with protease treatment and, in addition, is quite expensive; and general hESC suspension culture protocols have not yet been established. Moreover, it is also difficult to produce and expand hESCs that are of sufficient high quality for clinical applications through the use of suspension culture. Thus, many researchers still employ conventional feeder cell-based culture techniques for the study of embryonic stem cells.

Recently, Advanced Cell Technologies, Inc. (Marlborough, Mass) initiated an effort to cure macular dystrophy through the use of hESCs [14]. Even though the hESCs employed in this human clinical trial were established and maintained on mouse feeder cells, the culture system was approved because no contamination (i.e., mouse pathogens) originating from the feeder cells was detected. Regardless of this, the use of human feeder cells might be the best choice for the acceleration of clinical hESC therapies in the future. Previous studies have shown that hESCs can be successfully maintained on human feeder cell systems using human fibroblasts [15], [16], human mesenchymal cells [17], [18], and human placenta cells [19], [20] as the source of nutrient-affording cells. Recently, our laboratory successfully cultured hESCs and human induced pluripotent stem cells (iPSCs) on feeder cell layers composed of human adipose-derived stem cells (hASCs) [21], [22].

Representative feeder cell functions for the promotion of hESC stemness and expansion include the secretion of soluble factors and the provision of mechanical support [21], [23], [24]. For example, feeder cell-derived fibroblast growth factor (FGF)-2, transforming growth factor (TGF) ß-1, and activin-A are all critical soluble factors [21], [23] that sustain hESC stemness through ligand-receptor interactions [24]. Meanwhile, cell-matrix interactions between integrin in the hESC cell membrane and various ECM proteins (laminin, fibronectin, collagen and vitronectin) in the matrix of feeder cells feature predominantly in the mechanical support of the former by the latter [25], [26].

The type of intercellular connections between hESCs and feeder cells, and the roles that they play, have recently become the subjects of much investigation. Intercellular connections include desmosomes, tight junctions, adherent junctions, and gap junctions [27]. Among these, adherent junctions and gap junctions are essential for the promotion of hESC stemness and proliferation [28], [29], [30]. E-cadherin and connexin 43 (Cx43) are major protein constituents of adherent junctions and gap junctions, respectively, and are highly expressed in undifferentiated hESCs [28], [29], whereas downregulation of E-cadherin resulted in a loss of the undifferentiated state of hESCs [30]. Similar to E-cadherin expression, Cx43 expression was also dramatically reduced in differentiated hESCs compared with undifferentiated hESCs [31], [32].

Gap junctions are intercellular channels that are made up of two connexons. Each connexon is contributed by one of two adjoining cells and consists of six integral membrane proteins, termed connexins [33]. Gap junctional intercellular communication (GJIC) refers to the diffusion and exchange of intracellular molecules of less than 1.2 kD (i.e., small ions, second messengers, amino acids, metabolites, short interfering RNAs (siRNAs), and peptides) between neighbouring cells. Such intercellular coupling is implicated in the control of various cellular processes including cell proliferation, differentiation, migration, apoptosis, and metabolism [34], [35].

To date, 21 members of the connexin gene family have been identified in the human genome [36]. Of these, and as noted above, Cx43 is highly expressed in undifferentiated hESCs [37]. However, no information is available regarding GJIC between hESCs and human feeder cells. The purpose of this study was, therefore, to investigate whether GJIC exists between hESCs and hASC feeder cells, and to elucidate the relationship between GJIC and the stemness and proliferation of hESCs.

## Methods

### 2.1. Isolation of human ASCs

Human adipose stem cells (hASCs) was isolated from participants, who were provided to written informed consent for this study, under GMP conditions in the CHA Stem Cell Institute with approval from Institutional Review Board of CHA University Hospital Ethics Committee (IRB No. PBC09-099). hASCs were isolated from adipose tissue of a 56-year-old female, using a previously described method [38]. Briefly, the adipose tissues were washed at least three times with Dulbecco's phosphate-buffered saline (D-PBS, Wisent Inc., St-Bruno, Quebec, Canada) to remove blood. Adipose tissues were then digested in D-PBS containing 0.2% (w/v) bovine serum albumin (BSA) and 2 mg/ml collagenase type II (Sigma, St. Louis, MO, USA) for 45 min at 37°C, with intermittent shaking. After filtration through a 45 µm pore filter and centrifugation, floating adipocytes were removed from the stromal-vascular fraction. The hASCs isolated from the stromal-vascular fraction were cultured in Dulbecco's Modified Eagle Medium (DMEM, Gibco Invitrogen, Carlsbad, CA, USA) supplemented with 10% (v/v) fetal bovine serum (FBS, Gibco Invitrogen), 100 units/ml penicillin (Gibco Invitrogen), and 0.1 mg/ml streptomycin (Gibco Invitrogen) in humidified air with 5% (v/v) CO_2_ at 37°C.

### 2.2. Culture of hESCs

Undifferentiated hESCs (H9 cell line, WiCell Research Institute, Madison, WI) were mechanically propagated by micro-dissection on a monolayer of mitomycin C (MMC)-treated hASCs in Dulbecco's Modified Eagle's Medium (DMEM)/F12 medium (Gibco Invitrogen) supplemented with 20% serum replacement (SR, Gibco Invitrogen), 1% nonessential amino acids (Gibco Invitrogen), 1% penicillin-streptomycin (Gibco Invitrogen), 0.1 mM β-mercaptoethanol (Gibco Invitrogen), and 4 ng/ml basic fibroblast growth factor (bFGF) (Gibco Invitrogen) (hESC medium). The hASCs (1×10^4^ cells/cm^2^) were seeded onto 60 mm tissue culture dishes coated with 0.1% porcine gelatine, and cultured for 24 h. Following adhesion of hASC feeder cells to the 60 mm tissue culture dishes, hESCs colonies were re-plated onto freshly prepared hASC feeder cell layers. Following a subsequent 48 h incubation, the medium was refreshed every 24 h.

### 2.3. siRNA-mediated downregulation of Cx43

Cx43 RNA was downregulated in hESCs or hASCs by lipofection to introduce predesigned ON-TARGET plus SMARTpool™ siRNA constructs (Dharmacon, Chicago, IL, USA) targeted against Cx43. The ON-TARGET plus SMARTpool™ siRNA was purchased in an annealed and purified form that was ready to be transfected after re-suspension in water. For transfection, 2×10^4^ cells were plated onto 24-well plates and transfected at the time of plating with 50 nM siRNA, using DhamaFECT1 (Dharmacon), according to the recommendations of the manufacturer. Prior to use, hASCs and hESCs were exposed in the presence of siRNAs solution for 24 and 48 h, respectively.

### 2.4. Scrape loading/dye transfer assay

Gap junctional intercellular communication was determined by the scrape loading/dye transfer (SL/DT) assay as described previously [39] to investigate the transfer of the fluorescent dye Lucifer yellow from one cell into adjacent cells through functional gap junctions. Lucifer yellow is a fluorescent dye with a molecular weight of 457 Da. The dye is, therefore, unable to permeate the intact cell membrane. However, Lucifer yellow can diffuse from cell to cell through gap junction channels. For the scrape loading/dye transfer assay, hESCs (H9 cell line) in combination with hASCs or hASCs alone were cultured in 60 mm tissue culture dishes for 3 days. Cells were washed three times with CaMg-PBS, and incubated with 1 mg/ml Lucifer yellow CH (475 Da, Invitrogen) and 1 mg/ml Rhodamine-dextran (10 kDa, sigma-Aldrich) in CaMg-PBS. Rhodamine-dextran was used as a negative control dye to verify that Lucifer yellow dye-transfer occurs through functional gap junctions. Next, a scrape was quickly made with a sterile scalpel blade across the cell culture to allow the dye to be taken up by the wounded cells. After 3 min incubation, the dye was washed away using CaMg-PBS. Subsequently, the cells were fixed with 4% (v/v) formalin, and images were captured by using a fluorescence microscope to observe the spread of the dye from the wounded cells to the adjacent, intact cells.

### 2.5. Quantitative analysis of hESC colony size

The hESCs were grown for 5 days with control hASC feeder cells or hASC feeder cells treated with Cx43-siRNA. Microscopic (phase contrast) images were obtained, and the size of each hESC colony was quantitatively analysed by using the NIH Image J version 1.43u software (http://rsbweb.nih.gov/ij/) package, as follows. The average colony diameter (i.e., the average of the shortest and the longest colony diameter) was determined every day for 5 days for 20 hESC colonies per condition. The average colony diameter was then used to calculate the average colony area.

### 2.6. RNA extraction and quantitative real time-polymerase chain reaction (qRT-PCR) analysis

Total RNA was prepared from hESCs and hASCs by using TRIzol® (Gibco Invitrogen). Total RNA (1 µg) was used for cDNA synthesis, using a TOPscript^TM^ cDNA Synthesis kit (Enzynomics, Daejeon, Korea). qRT-PCR reactions were set-up in a total volume of 20 µl with AccuPower GreenStar qPCR-PreMix (Bioneer) and 1 µl forward and reverse primers. A Exicycler^TM^ 96 Superior 5-color Real-Time Quantiative PCR system (Bioneer) was used to run the samples, with initial denaturation step was performed at 95°C for 5 min, then 45 cycles of 10 s at 95°C and 45 s at 55°C, follwed by a melt curve. Data was analysed using the 2^−ΔΔct^ method. Values were analysed using GAPDH as a housekeeping gene and normalized relative to control, with standard deviation (Table 1). All qRT-PCR experiments were performed in quadruplicate, and carried out at least three independent experiments.

**Table 1:** Nucleotide sequences of human-specific primer sets.

Primer name	Sequences		Accession number	Product size (bp)
*OCT*4	F	5′- AGT GAG AGG CAA CCT GGA GA- 3′	NM_002701.4	110 bp
	R	5′- ACA CTC GGA CCA CAT CCT TC -3′		
*SOX*2	F	5′- TGG ACA GTT ACG CGC ACA T -3′	NM_003106.3	138 bp
	R	5′- TCA CGT CGT AGC GGT GCA T -3′		
*NANOG*	F	5′- CAT GAG TGT GGA TCC AGC TTG- 3′	NM_024865.2	141 bp
	R	5′- TGA GGC ATC TCA GCA GAA GAC- 3′		
*Cx*43	F	5′- CAC ACT CTT GTA CCT GGC TCA-3′	NM_000165.3	101 bp
	R	5′- TGA CAC CAT CAG TTT GGG CA -3′		
*GAPDH*	F	5′- ACA TCG CTC AGA CAC CAT G -3′	NM_002046.3	143 bp
	R	5′- TGT AGT TGA GGT CAA TGA AGG G-3′		

Abbreviations: Cx, connexin; GAPDH, glyceraldehyde-3-phosphate dehydrogenase.

### 2.7. Western blot analysis

Cells were lysed in RIPA buffer consisting of 150 mM NaCl, 50 mM Tris-HCl (pH 8.0), 5 mM EDTA, 0.5% sodium deoxycholate, 0.1% sodium dodecyl sulphate (SDS), 20 M leupeptin, 5 g/ml pepstatin, and 1 mg/ml aprotitin (Sigma). After sonication and centrifugation at 10 000 rpm for 10 min, protein concentrations were measured by using the Bradford method. Equal amounts of cell extract were separated in a 10% SDS-bisacrylamide gel and then transferred onto a polyvinylidene fluoride (PVDF) membrane using the Trans-Blot semi-dry transfer kit (Bio-Rad, Hercles, CA, USA). The membranes were blocked with 5% non-fat milk for 1 h and probed with anti-Cx43 (1∶500; Millipore, Billerica, MA, USA), anti-OCT4 (1∶1000; Abcam, Cambridge, MA), anti-SOX2 (1∶1000; Abcam), anti-NANOG (1∶1000; Abcam), and anti-α-tubulin (1∶1000, Santa Cruz, CA) primary antibodies. The blots were then incubated with horseradish peroxidase-conjugated secondary antibodies (1∶10 000, Sigma), and immunoreactive bands were visualised using the WEST-one Western blotting detection system (iNtRON Biotechnology, Kyungki-Do, Korea). All western blotting experiments were performed in quadruplicate, and carried out at least three independent experiments.

### 2.8. Immunocytochemistry and AP staining

For immunocytochemistry analyses, hESCs and hASCs were fixed with 4% paraformaldehyde in PBS for 20 min, permeabilised with 0.2% Triton X-100 in PBS for 10 min, and blocked with 2% BSA in PBS for 30 min. Afterwards, the cells were treated with primary antibodies against Cx43 (1∶100; Abcam) and OCT4 (1∶1000; Abcam) to observe hESC stemness. Cy3-and FITC-conjugated secondary antibodies were used to detect and visualise the primary Cx43 and OCT4 antibodies. All antibodies were diluted in 1% BSA solution. Alkaline phosphatase (AP) staining was also performed using the BCIP/NBT Liquid Substrate System (Sigma).

### 2.9 Karyotype analysis

For karyotype analysis, hESCs were cultured with control or Cx43-siRNA-treated hASC feeder cells and maintained in DMEM/F12 supplemented with 20% SR and 4 ng/mL bFGF for 5 days. hESC colonies were incubated with colcemid solution (10 µg/ml, Invitrogen) in hESC medium for 3 h at 37°C in the presence of 5% CO_2_. The colcemid-treated cells were washed with PBS and treated with a hypotonic solution (1% sodium citrate in distilled water; Sigma) for 30 min at 37°C. The cells were then dispersed by trypsin treatment and collected by centrifugation. Lysed cells were fixed in a 3∶1 solution of methanol/acetic acid. G banding was analysed for the identification of chromosomes.

### 2.10. Confocal laser scanning microscope imaging

Immunofluorescence images of hESCs and hASCs were taken on a confocal laser scanning microscope (CLSM; Carl ZEISS, LSM 510 Meta, Jena, Germany).

### 2.11. Statistical analysis

Each experiment was performed at least in quadruplicate. Quantitative data are expressed as the mean ± the SD. Repetitive analysis of variance (ANOVA) was used for analysis of quantitative values, and the Tukey's post hoc test (inerSTAT-a v1.3) was used for all pair-wise comparisons among groups. A P-value of less than 0.01 was considered statically significant.

## Results

### 3.1. Expression of Cx43 in hESCs and hASCs

**Figure 1:**
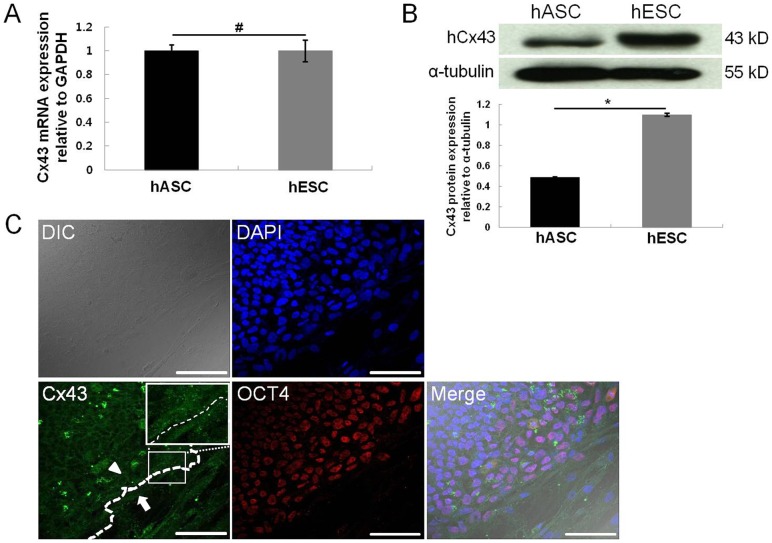
Expression of Cx43 in hESCs and hASCs. The expression of endogenous Cx43 in hESCs and hASCs was confirmed by qRT-PCR (A) and Western blot analysis (B). Expression of OCT4 (red) and Cx43 (green) in hESCs and hASCs was observed by immunocytochemistry (C). Nuclei were counterstained with DAPI (blue). The arrow head indicates the expression of endogenous Cx43 in hESCs adjacent to hASCs (arrow). The white box indicates the Cx43 expression at the border line. All data are shown as the mean ± the standard deviation (SD) (n = 4; ^#^, p>0.05; *, p<0.05). Scale bar, 100 μm.

The expression of Cx43 in hESCs and hASCs was investigated by qRT-PCR and Western blot analysis (Fig. 1A and 1B). Both cell types expressed Cx43 at the mRNA and protein levels (Fig. 1A and 1B). However, the expression of Cx43 protein was 2.3 fold much higher in hESCs than in hASCs (Fig. 1B). This result is coincident with previous reports, demonstrating that Cx43 was enriched in undifferentiated hESCs compared with differentiated embryoid bodies and adult human tissues. Immunocytochemical results (Fig. 1C) showed that the border line between hESCs and hASCs in co-culture was distinguished by the expression of OCT4 protein. Furthermore, the protein expression of Cx43 was also confirmed in both hESCs and hASCs. Cx43 expression was largely found in intercellular regions, and was higher in hESCs relative to hASCs. Interestingly, Cx43 expression, like OCT4 expression, was clearly observed at the border line between hESCs and hASCs.

### 3.2. Evidence of gap junction formation between hESCs and hASCs

**Figure 2:**
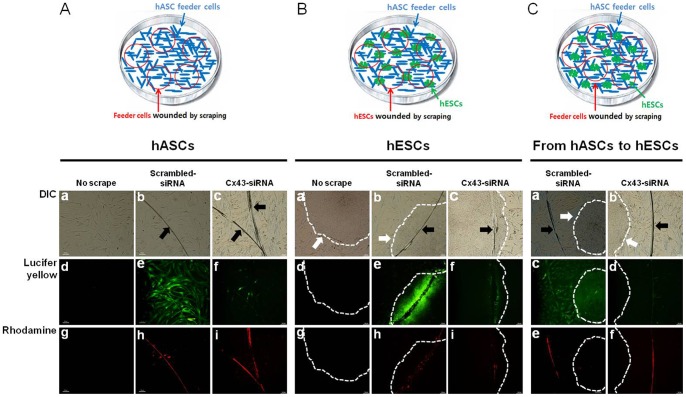
GJIC between hESCs and hASCs. Functional gap junction channels were detected by the scrape loading/dye transfer assay for hASCs cultured alone and with hESCs. Phase contrast (A(a-c), B(a-c), C(a,b)), Lucifer yellow (A(d-f), B(d-f), C(c,d)) and rhodamine-dextran (A(g-i), B(g-i), C(e,f)) of hASCs and hESCs are shown. A scrape was made across the monolayer to allow the uptake of Lucifer yellow by the wounded cells. The dye was then transferred from the wounded cells to the adjacent, intact cells via functional gap junction channels. Lucifer yellow dye transfer was shown from hASCs to adjacent hASCs (A(e,f)); hESCs to adjacent hESCs (B(e,f); and hASCs to adjacent hESCs (C(c,d)). No-scrape group was employed as the normal conditions (A(d) and B(d)). Rhodamine-dextran was used as a negative control, showing no dye transfers from the wounded cells to neighbouring cells (A(h,i), B(h,i), C(e,f)). The black and white arrows indicate the scraped cells and the hESC colonies, respectively. The white dotted lines show the boundary between the hASCs and hESC colony. Scale bar, 100 μm.

To identify functional gap junction channels in hESCs and hASCs, and between hESC and hASCs, a scrape loading/dye transfer assay with Lucifer yellow and rhodamine-dextran was performed. Because of its low molecular weight (457 Da), Lucifer yellow dye transfers from wounded cells to neighbouring cells via functional gap junction channels. Meanwhile, rhodamine-dextran (10,000 Da) is too large molecule to transfer through functional gap junction channels, thereby being used as a negative control to confirm that the Lucifer yellow dye transfer was solely caused by gap junction coupling and not caused by cytoplasmic bridges or membrane fusions. As shown in Fig. 2, Lucifer yellow dye diffusion illustrating GJIC was observed between both hASCs and hESCs (Fig. 2A(e) and 2B(e)). However, Cx43-siRNA treated cells showed a dramatic decrease in Lucifer yellow dye diffusion (Fig. 2A(f) and 2B(f)). No dye transfer occurred under normal conditions (no scrape, Fig. 2A(d) and 2B(d)). We also examined intercellular communication through functional gap junction channels between hESCs and hASCs. Lucifer yellow dye spread was observed to spread from wounded hASCs to adjacent hESCs via functional gap junction channels (Fig. 2C(c)), whereas Cx43-siRNA treated hASCs showed no dye transfers from wounded hASCs to adjacent hESCs (Fig. 2C(d)). These results suggest that functional GJIC occurred between hESCs and hASC feeder cells.

### 3.3. Involvement of Cx43 in hESC stemness

**Figure 3:**
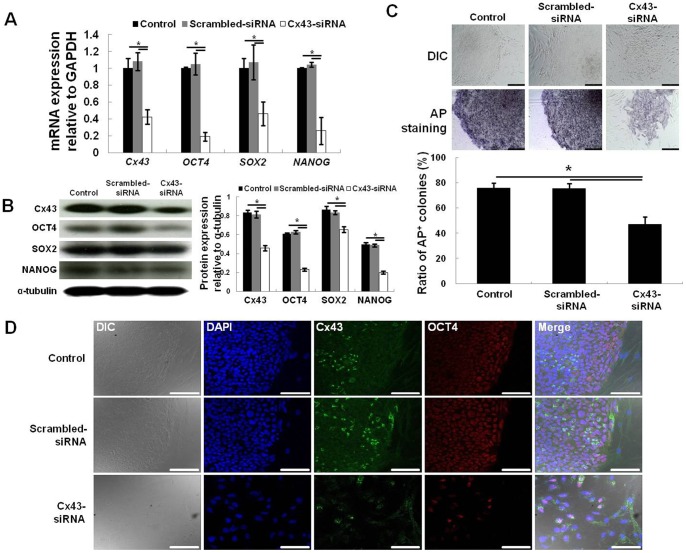
Effect of Cx43 downregulation on hESC stemness. Downregulation of Cx43 and stemness gene (*OCT4*, *SOX2*, and *NANOG*) expression in Cx43-siRNA-treated hESCs was confirmed by qRT-PCR after 2 days culture (A) and Western blot analysis after 4 days culture (B). The percentage of AP-positive colonies formed by hESCs following Cx43-siRNA treatment was significantly lower than the percentage of AP-positive colonies formed by hESCs following control or scrambled-siRNA treatment (C). Immunocytochemical analysis demonstrated that OCT4 and Cx43 protein expression was reduced in Cx43-siRNA-treated hESCs versus control or scrambled-siRNA-treated hESCs (D). (C) and (D) were evaluated after 5 days culture. All data are shown as the mean ± the SD (n = 4; *, p<0.05). Scale bar, 100 μm.

To confirm the effect of Cx43 downregulation on hESCs stemness, hESCs were treated with Cx43-siRNA or scrambled-siRNA in hESC medium. The effect of siRNA treatment was then investigated by qRT-PCR and Western blot analysis (Fig. 3A and 3B). Compared with control hESCs (no treatment), Cx43-siRNA-treated hESCs showed a dramatic decrease in target Cx43 gene expression (>50%), as well as stemness gene expression (*OCT4*, *SOX2*, *NANOG*, >50%). Meanwhile, treatment of hESCs with scrambled-siRNA did not alter gene expression of Cx43 or stemness genes (*n* = 4; *, p<0.05; Fig. 3A). As shown in Fig. 3B, Cx43-siRNA-treated hESCs also exhibited a lower level of protein expression for Cx43 and stemness markers (OCT4, SOX2, and NANOG) relative to control and scrambled-siRNA-treated hESCs (*n* = 4; *, p<0.05).

After 5 days in culture, the percentage of AP-positive hESC colonies among 100 colonies was evaluated. The percentage of AP-positive colonies for Cx43-siRNA-treated hESCs (47+/−6.1%) was significantly decreased relative to the number of AP-positive colonies for control and scrambled-siRNA-treated hESCs (76+/−4% and 75+/−3.9%, respectively) (*n* = 4; *, p<0.05; Fig. 3C). Furthermore, the Cx43-siRNA-treated hESC colonies did not maintain the characteristics of a round and compact colony morphology followed by a decrease in the expression of stemness markers that were exhibited by the other colonies (Fig. 3D). This result demonstrates that Cx43-siRNA successfully targeted and decreased Cx43 gene expression in hESCs, resulting in a decrease in their stemness.

### 3.4. Downregulation of Cx43 in hASCs by siRNA treatment

**Figure 4:**
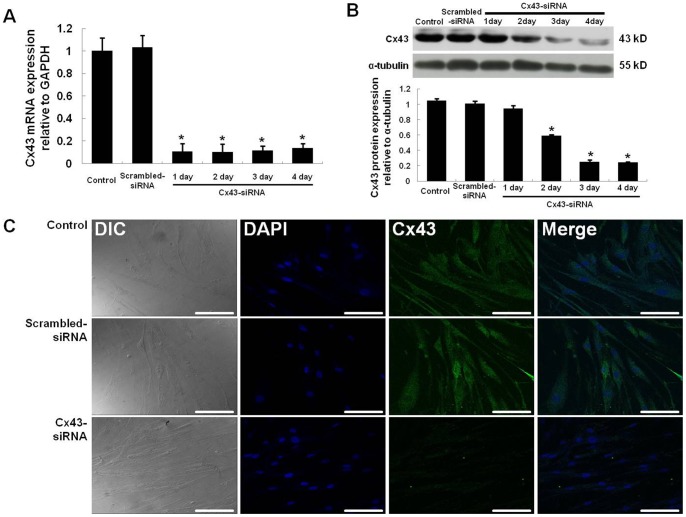
siRNA-mediated downregulation of Cx43 in hASCs. The time-dependent, siRNA-mediated downregulation of Cx43 in hASCs was investigated by qRT-PCR (A) and Western blot analysis (B). Immunocytochemical analysis confirmed the Cx43 downregulation in hASCs after 3 days of siRNA treatment (C). Nuclei were counterstained with DAPI. All data are shown as the mean ± the SD (n = 4; *, p<0.05). Scale bar, 100 μm.

To downregulate Cx43 expression in hASCs, cells were treated with scrambled-siRNA or Cx43-siRNA and cultured for 4 days. According to qRT-PCR and Western blot analysis, each group of hASCs expressed Cx43 mRNA and protein (*n* = 4; *, p<0.05; Fig. 4A and B). Compared with control hASCs (no treatment) and scrambled-siRNA-treated hASCs, Cx43-siRNA-treated hASCs exhibited distinctly lower levels of Cx43 mRNA and protein on day 1 of culture; these levels progressively decreased over the 4 days in culture. In addition, Cx43 protein was detected via immunocytochemistry in control and scrambled-siRNA-treated hASCs, but not in Cx43-siRNA-treated hASCs (Fig. 4C). Dissimilar from hESCs, knockdown of Cx43 did not alter the morphology of hASCs. Thus, Cx43-siRNA treatment successfully targeted and eliminated gene and protein expression of Cx43 in hASCs.

### 3.5. Stemness and growth of hESCs on Cx43-siRNA-treated hASCs

**Figure 5:**
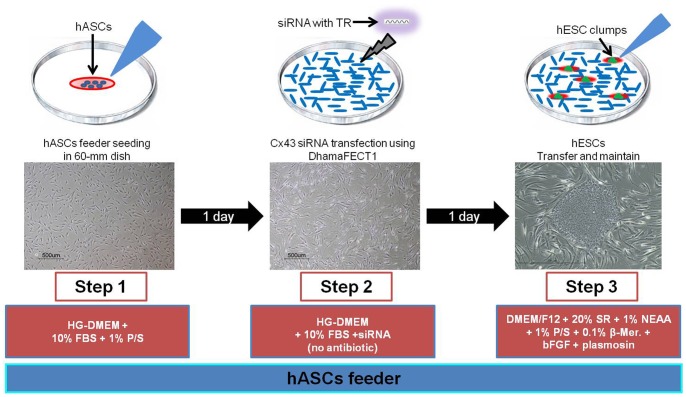
Schematic diagram of Cx43-siRNA treatment method. To downregulate Cx43 expression in hASC feeder cells, hASCs were seeded at 2.1×10^5^ cells per 60 mm tissue culture plate before 24 h. hASCs were washed with PBS and then treated with scrambled-siRNA or Cx43-siRNA and cultured for 24 h. Twenty-four hours after transfection, hESCs clumps were transferred onto siRNA treated hASC feeder cells and maintained for 5 days.

**Figure 6:**
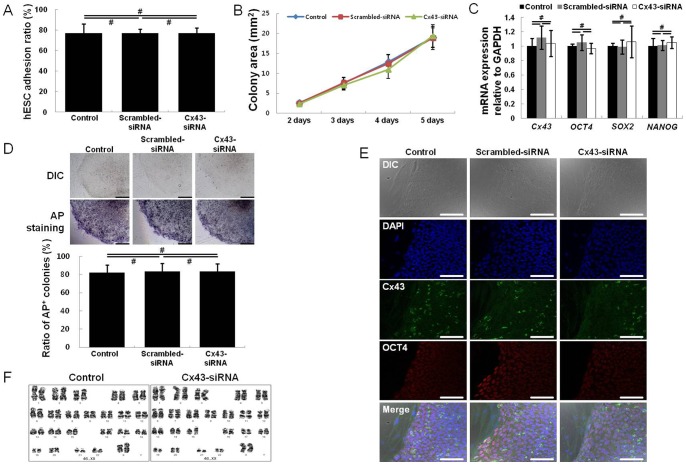
Culture of hESCs on Cx43 siRNA-treated hASCs. Compared with treatment of hASCs with control or scrambled-siRNA, the Cx43-siRNA treatment of hASC feeder cells did not alter adhesion (A), colony growth (n = 20; B), gene expression (i.e., *OCT4*, *SOX2*, and *NANOG*) (C), AP-positive colony numbers (D), OCT4 expression (E), or chromosomal stability (F) of co-cultured hESCs. Nuclei were counterstained with DAPI (E). All data are shown as the mean ± the SD. (n = 4; ^#^, p>0.05). Scale bar, 100 μm.

Next, we investigated the importance of a direct Cx43-mediated interaction between hESCs and hASCs for hESC stemness and growth. To do this, hESCs were cultured on Cx43-siRNA-treated hASCs. The siRNA-treated feeder cells were prepared by culturing hASCs with siRNA for 1 day. Subsequently, the hESCs were seeded on the siRNA-treated hASCs and then cultured for 5 days (Fig. 5). During the 5 day culture period, both hASCs and hESCs maintained their characteristic morphologies (fibroblastic shape and round colony formation, respectively). The initial adhesion of hESC colonies to control (no treatment), scrambled-siRNA-treated and Cx43-siRNA-treated hASCs was examined after 5 days of culture (Fig. 6A). Adhesion to the three groups of hASCs did not significantly differ; the values were 82+/−7%, 80+/−5%, and 80+/−9% for control, scrambled-siRNA-treated, and Cx43-siRNA-treated hASCs, respectively (*n* = 4; ^#^, p>0.05; Fig. 6A). In addition, the average area of 20 hESC colonies cultured on each hASC substrate was employed as a measure of hESC growth and did not significantly differ between the hASC groups (*n* = 20; ^#^, p>0.05; Fig. 6B). These results demonstrate that Cx43-mediated GJIC between hESCs and hASCs was not an important factor for the initial adhesion and subsequent growth of hESCs on hASC feeder cells.

As shown in Fig. 6C, hESCs cultured on each group of hASCs showed no difference in the gene expression level of Cx43 or the stemness genes (*OCT4*, *SOX2,* and *NANOG*), even after 10 passages (*n* = 4; ^#^, p>0.05). Immunocytochemistry confirmed that protein expression levels of Cx43 and OCT4 were unaltered in hESCs cultured on Cx43-siRNA-treated compared with control and scrambled-siRNA-treated hASCs (Fig. 6E). Furthermore, most of the hESC colonies demonstrated strong expression of AP (indicative of pluripotency), irrespective of siRNA treatment (Fig. 6D, top image). To quantify the pluripotent hESC colonies, the number of AP-positive colonies among 100 hESC colonies was counted. The percentage of AP-positive hESC colonies for the Cx43-siRNA treated hASC group (82+/−8%) was not significantly different from the percentage of AP-positive colonies for the control (84+/−8%) or the scrambled-siRNA-treated hASC group (83+/−9%) (*n* = 4; ^#^, p>0.05) (Fig. 6D, bottom images). Furthermore, hESCs cultured on Cx43-siRNA-treated hASCs exhibited a normal karyotype of 46, XX after 10 passages (Fig. 6F). Hence, Cx43-mediated GJIC between hESCs and hASC feeders was also not required for the maintenance of hESC stemness.

## Discussion

This study demonstrated that functional GJIC occurs between hESCs and hASC feeder cells. While many previous studies have focused on hESC-matrix interactions, the current study is the first to report intercellular communication between hESCs and human feeder cells. Present study has focused on Cx43, a major gap junctional protein, even though there is a possibility of other types of connexin between hESCs and human feeder cells [40], [41]. We hypothesized Cx43 gap junction communication between hESC and human feeder cells would be an important factor for stemness and expansion. However, we found that such intercellular communication was not a prerequisite for the support of hESC stemness and growth by hASCs.

Generally, gap junction-mediated coupling is highly functional at the ICM in early human embryos [42]. Undifferentiated hESCs express Cx43 and communicate with adjacent hESCs via GJIC [28], [31], [43]. Interestingly, hASCs also express Cx43 and can communicate with each other via gap junctions [44]. Our results (Fig. 1) are therefore consistent with previous reports showing Cx43 expression in both hESCs and hASCs.

Recently, Galat *et al.* demonstrated that Cx43 expression was decreased at the border between undifferentiated and differentiated cells in hESC colonies [32]. Assou *et al.* compared 38 microarray data points in undifferentiated hESCs and differentiated hESCs, and concluded that Cx43 could be an alternative stemness marker for hESCs [45]. Moreover, Todorova et al. reported that downregulation of Cx43 in mouse ESCs was attenuated during proliferation and maintenance of stemness [46]. We also found that the expression of hESC stemness markers (OCT4, SOX2, and NANOG) was downregulated by the treatment of hESCs with Cx43-siRNA (Fig 3). In addition, the size of AP-positive hESCs colonies was significantly diminished following Cx43-siRNA treatment, and the constituent cells assumed the morphology of differentiated cell types. These results indicate that Cx43 expression in hESCs is critical for the preservation of their stemness.

Studies of various cell and tissue types have shown that the exchange of small molecules between cells via GJIC is essential for the regulation of proliferation, differentiation, and apoptosis [47], [48]. For the detection of functional GJIC, scrape loading and dye transfer (SL/DT) is a fast and simple technique in wide use even though microinjection technique allows the observation of GJIC in single cell level [39], [49]. This SL/DT technique typically employs a layer of cells that is wounded by mechanical scraping. If the cells are connected via functional gap junctions, the loading dye (e.g., Lucifer yellow, 457 Da) absorbed by the wounded cells diffuses away and enters the neighbouring cells. This methodology was successfully used by Raymond et al. to establish GJIC between hESCs [50]. By the SL/DT method, we also observed active GJIC between hESCs (Fig 2). Interestingly, Lucifer yellow transfer between hASCs was also found, although the dye transfer was not as extensive as that between hESCs. Our observations are consistent with previous work demonstrating that mesenchymal stem cells contain functional gap junctions [44], [51].

Wong et al. showed that there is no communication between hESC and the feeder layer of MEF [50]. However, Huettner et al. found gap junction-mediated coupling between hESCs and mouse fibroblast feeder cells even though not in many cases [52]. The most of previous studies were achieved between hESCs and mouse feeder cells, not human feeder cells. Moreover, there is no report to investigate GJIC between hESCs and human feeder cells by regulating gap junction proteins such as Cx43 directly.

The current study focused on GJIC between hESCs and human feeder cells. Surprisingly, the SL/DT assay provided evidence for active GJIC between hESCs and hASCs in co-culture, as well as between hESCs and hASCs in mono-culture. This clearly illustrates that hESCs and hASCs can communicate via gap junctions. By contrast, James et al. reported that GJIC rarely occurred between hESCs and mouse feeder cells, as determined by the microinjection and transfer of Lucifer yellow [52]. The contradictory results of the James et al. study and our own study might be due to different experimental conditions, such as mouse rather than human feeder cell lines.

To evaluate the effect of Cx43-mediated GJIC between hESCs and hASC feeder cells on hESC stemness and growth, control hESCs were cultured on Cx43-siRNA-treated hASCs. Cx43-siRNA treatment distinctly downregulated Cx43 expression in hASCs (Fig. 4), but hESCs in co-culture exhibited normal proliferation (colony growth) and stemness (AP, *OCT4*, *SOX2*, and *NANOG* expression) (Fig 6). These results were also reconfirmed by stable Cx43-shRNA (Figures S1-S4). Moreover, the hESCs cultured on Cx43-siRNA-treated hASCs exhibited a normal karyotype, even after 10 hESC passages. Thus, GJIC between hESCs and hASCs is not required for the proliferation and stemness of hESCs.

In conclusion, this study confirmed that Cx43-mediated GJIC between hESCs is critical for the maintenance of hESC stemness during expansion. This study also found that hESCs can communicate with hASC feeder cells through functional gap junction channels. However, GJIC between hESCs and ASCs is not essential for the preservation of hESC stemness and the promotion of hESC proliferation. Additional factors including growth factors (i.e., FGF-2, Activin-A, TGF-1, etc.) and ECM molecules secreted by feeder cells, therefore, are anticipated to be more important than hESC-hASC GJIC for the regulation of crucial hESC functions.

## Supporting Information

Figure S1 **Downregulation of Cx43 in hASCs by shRNA treatment.** For downregulation of Cx43, pTRIPZ lentiviral vector with doxycycline inducible shRNA and RFP was transducted into hASCs. The downregulation of Cx43 in hASCs was induced by doxycycline and confirmed by qRT-PCR (A) and Western blot analysis (B). The inducible expression of RFP was detected in hASCs after doxycycline treatment by fluorescence microscope (C). All data are shown as the mean ± the SD. (n = 4; ^*^, p<0.05). Scale bar, 100 μm.
(TIF)

Figure S2 **Culture of hESCs on Cx43-shRNA-treated hASCs feeder.** The hESCs cultured on Cx43-shRNA-treated hASCs feeder showed no difference in the cellular morphology compared with those on control or scrambled-shRNA hASCs feeders. Scale bar, 100 μm.
(TIF)

Figure S3 **Stemness of hESCs on Cx43-shRNA-treated hASCs feeder.** Compared with control or scrambled-shRNA hASCs feeder, Cx43-shRNA-treated hASCs feeder did not alter the expression level of genes (A) and proteins (B) (i.e., OCT4, SOX2, and NANOG), and AP-positive colony numbers (C) associated with stemness of hESCs. All data are shown as the mean ± the SD. (n = 4; ^#^, p>0.05). Scale bar, 100 μm.
(TIF)

Figure S4 **Apoptotic quantification of hESCs on Cx43-shRNA-treated hASCs feeder.** Apoptotic cells are quantified by flow cytometry analysis after staining with Annexin V and propodium iodide (PI). The hESCs on control, scrambled-shRNA and Cx43-shRNA-treated hASCs feeder showed 10–15% apoptotic level with no significance. All data are shown as the mean ± the SD. (n = 5; ^#^, p>0.05).
(TIF)

Methods S1 **Supplementary Methods.**
(DOCX)
