# Supplementary figures and images for: hESC Expansion and Stemness Are Independent of Connexin Forty-Three-Mediated Intercellular Communication between hESCs and hASC Feeder Cells
Source: PLoS One. 2013 Jul 26;8(7):e69175. doi: 10.1371/journal.pone.0069175 (PMC3724839; doi:10.1371/journal.pone.0069175)

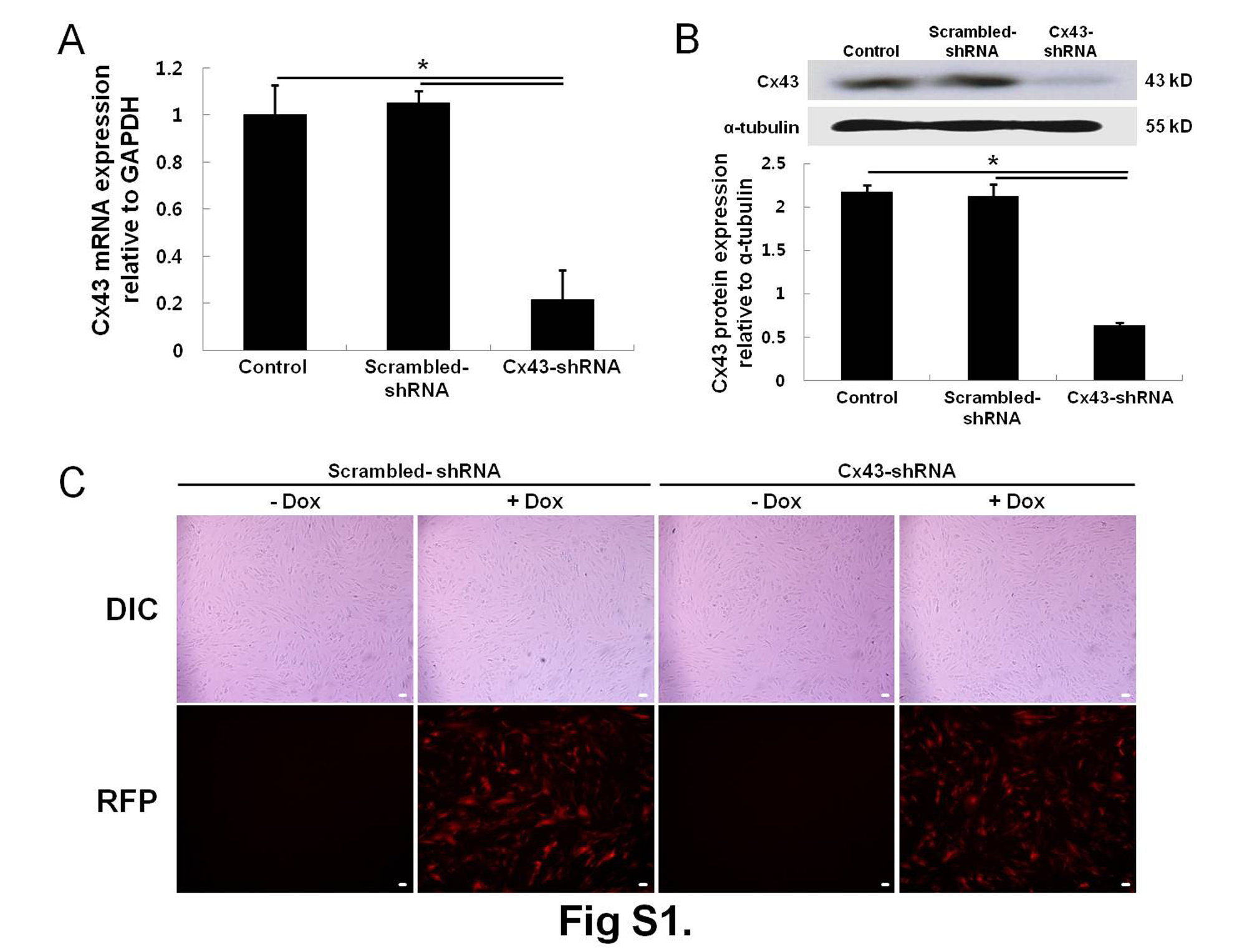

Supplement: Figure S1 — Downregulation of Cx43 in hASCs by shRNA treatment. For downregulation of Cx43, pTRIPZ lentiviral vector with doxycycline inducible shRNA and RFP was transducted into hASCs. The downregulation of Cx43 in hASCs was induced by doxycycline and confirmed by qRT-PCR (A) and Western blot analysis (B). The inducible expression of RFP was detected in hASCs after doxycycline treatment by fluorescence microscope (C). All data are shown as the mean ± the SD. (n = 4; *, p<0.05). Scale bar, 100 μm. (TIF) [file pone.0069175.s001.tif]

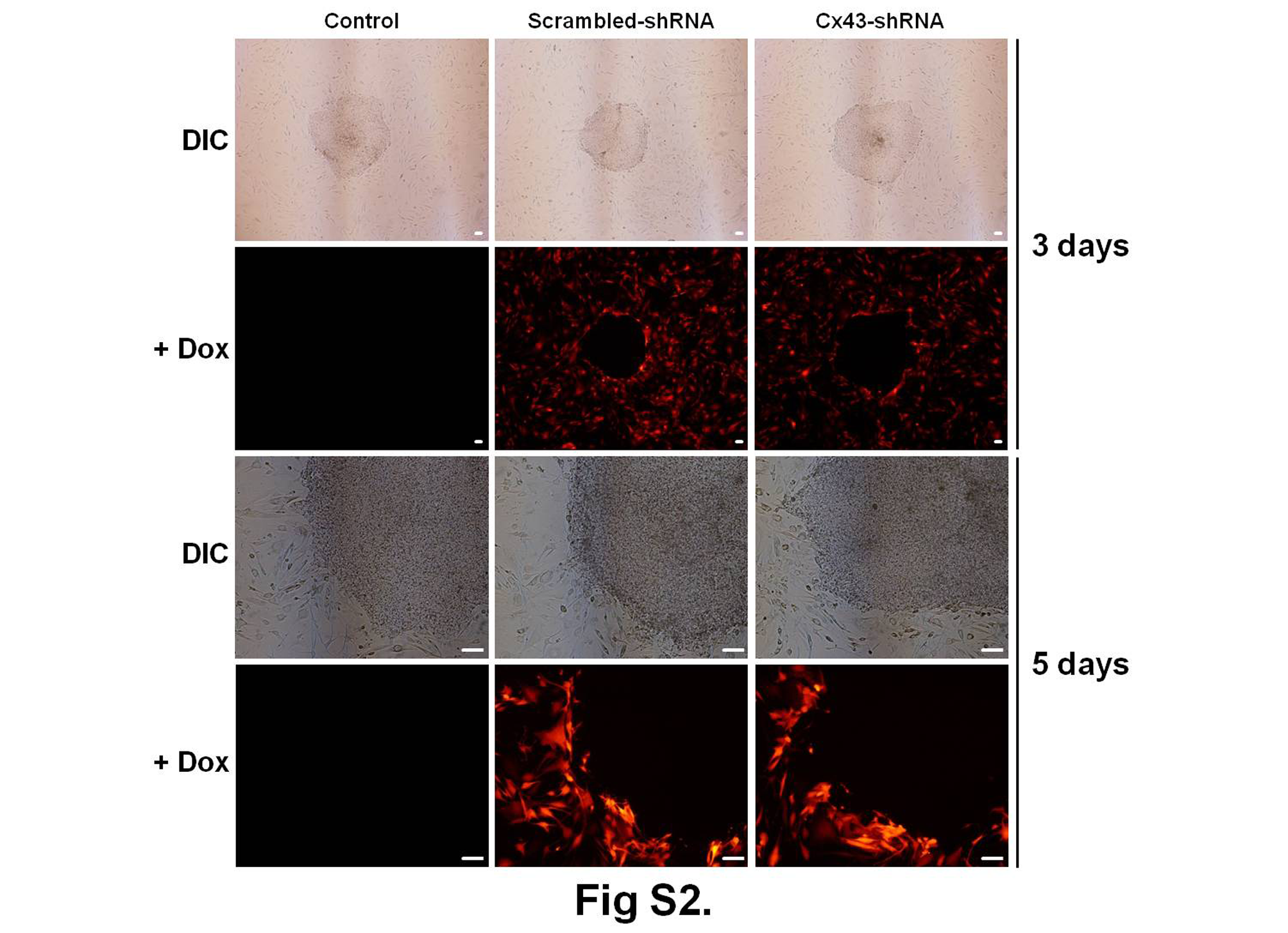

Supplement: Figure S2 — Culture of hESCs on Cx43-shRNA-treated hASCs feeder. The hESCs cultured on Cx43-shRNA-treated hASCs feeder showed no difference in the cellular morphology compared with those on control or scrambled-shRNA hASCs feeders. Scale bar, 100 μm. (TIF) [file pone.0069175.s002.tif]

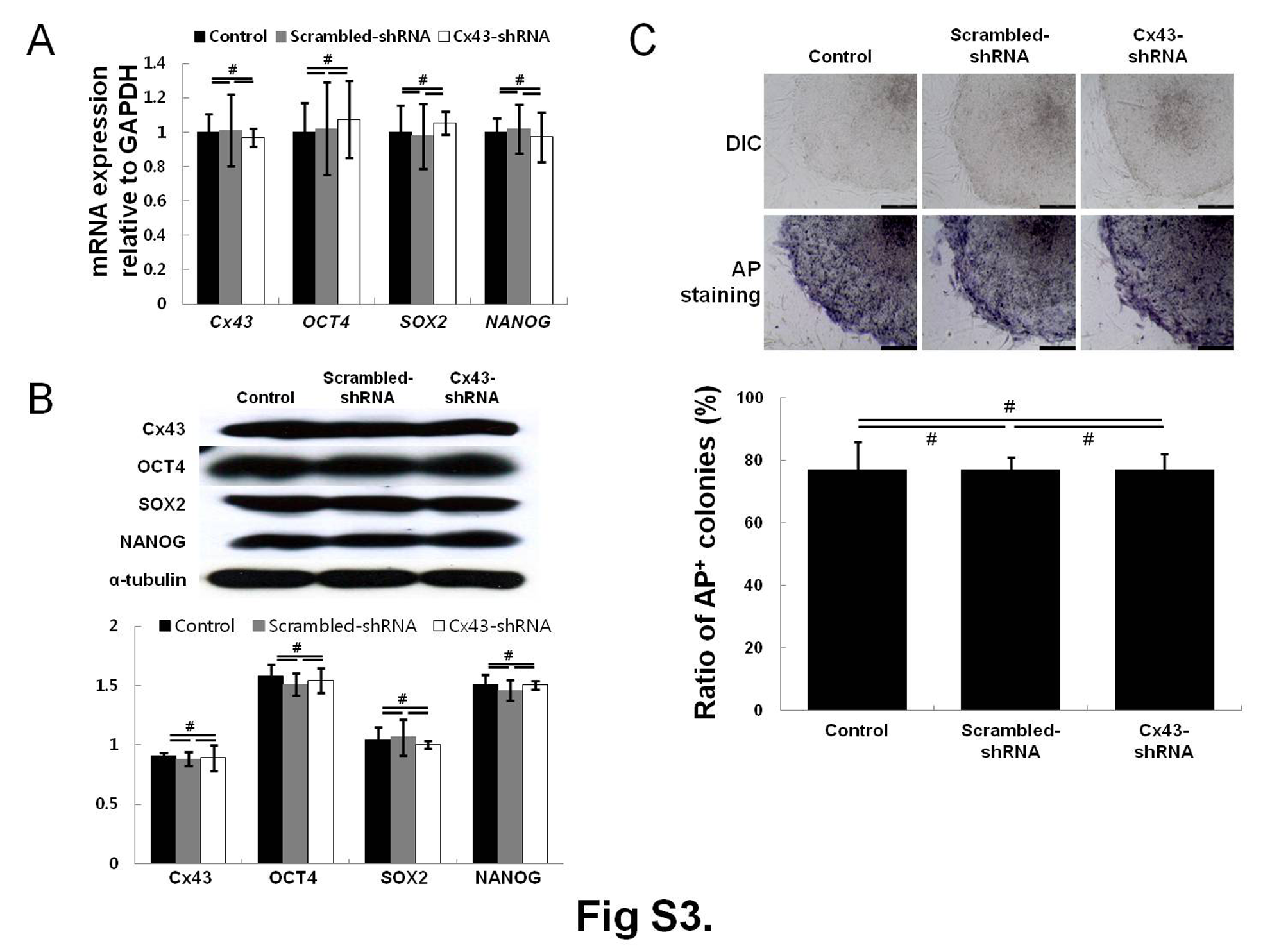

Supplement: Figure S3 — Stemness of hESCs on Cx43-shRNA-treated hASCs feeder. Compared with control or scrambled-shRNA hASCs feeder, Cx43-shRNA-treated hASCs feeder did not alter the expression level of genes (A) and proteins (B) (i.e., OCT4, SOX2, and NANOG), and AP-positive colony numbers (C) associated with stemness of hESCs. All data are shown as the mean ± the SD. (n = 4; #, p>0.05). Scale bar, 100 μm. (TIF) [file pone.0069175.s003.tif]

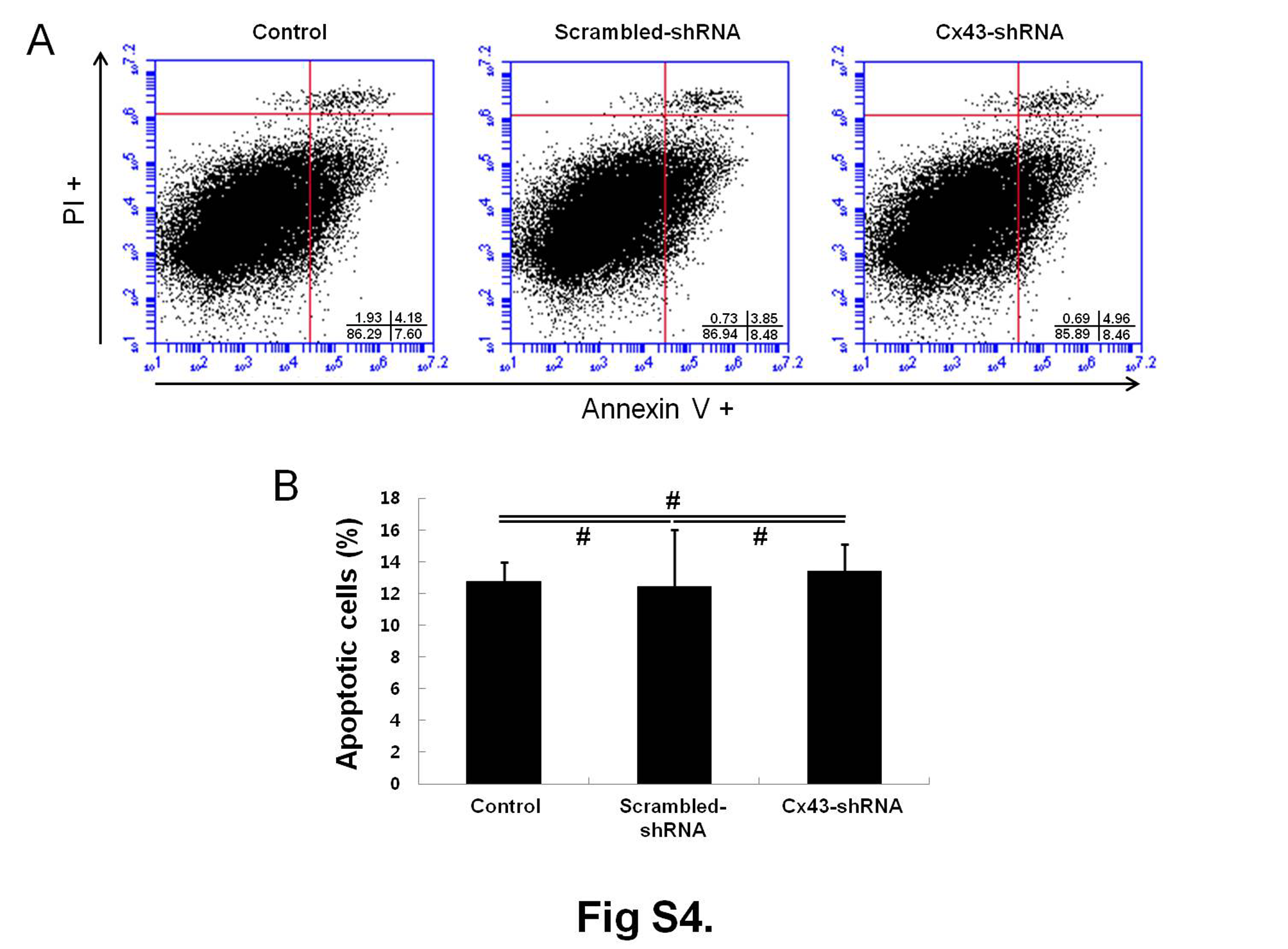

Supplement: Figure S4 — Apoptotic quantification of hESCs on Cx43-shRNA-treated hASCs feeder. Apoptotic cells are quantified by flow cytometry analysis after staining with Annexin V and propodium iodide (PI). The hESCs on control, scrambled-shRNA and Cx43-shRNA-treated hASCs feeder showed 10–15% apoptotic level with no significance. All data are shown as the mean ± the SD. (n = 5; #, p>0.05). (TIF) [file pone.0069175.s004.tif]
