# Supplementary material for: hESC Expansion and Stemness Are Independent of Connexin Forty-Three-Mediated Intercellular Communication between hESCs and hASC Feeder Cells
Source: PLoS One. 2013 Jul 26;8(7):e69175. doi: 10.1371/journal.pone.0069175 (PMC3724839; doi:10.1371/journal.pone.0069175)
Supplement: Methods S1 — Supplementary Methods. (DOCX) [file pone.0069175.s005.docx]

**Methods S1**

**1.1. Lentivirus production and infection**

Lenti-X 293T cells (Thermo Fisher Scientific, Logan, UT) were plated at 7 x 10^6^ cells per 100 mm tissue culture plate in growth medium and incubated at 37 °C, 5% CO2 incubator overnight. Diluted 20 μg plasmid DNA (pTRIPZ) and 36 μl lentiviral packaging mixed up to 600 μl of X-fect reaction buffer (Clontech) in a micro tube and then polymer 9 μl was added into mixture tube, according to the manufacturer's instructions. Forth-eight hours after transfection, the supernatant of transfectant was collected and filtered through a 0.45 μm pore-size filter (Whatman, Maidstone, UK). For lentivirus transduction, hASCs were seeded at 100 mm tissue culture plate before 24 h for transduction. hASCs were washed with PBS and then replaced with virus-containing supernatant supplemented with 8 μg/ml polybrene (sigma), and subsequently incubated for 24 h.

**1. 2. Selection of stable shRNA cells**

For selection of stable shRNA expressed cell line, shRNA treansfected hASCs were washed with PBS and then 0.5 μg/ml puromycin added to the growth medium. Puromycin containing medium was refreshed every 2 days and the cells were incubated until 7 days.

**1. 3. Induction of TurboRFP/shRNA expression**

Puromycin-resistant stable hASCs were washed with PBS and then medium containing doxycycline was added at a concentration of 2 μg/ml. Following a subsequent 24 h incubation, the medium containing doxycycline was refreshed every 24 h. The addition of doxycycline induced expression of TurboRFP and Cx43-shRNA in TRIPZ vectors with a Tet-on configuration. TurboRFP expression was detected after 3 or 5 days of the induction using fluorescent microscope (Nikon, TS1500, Japan). The mRNA and protein of Cx43 in the cells was analysed by qRT-PCR and Western blotting, respectively.

**1. 4. Apoptosis analysis**

The level of apoptosis in hESCs was quantified with FACS analysis using FITC Annexin V apoptosis detection kit with PI (Biolegend, San Diego, CA), according to the manufacturer's instructions. The 5 x 10^5^ cells were washed twice with PBS, re-suspended in 40 μl of Annexin V binding buffer and then transferred in a 5 ml test tube. The stock solution of 5 μl Annexin V-FITC and 10 ul propidium iodide (PI) was added into the test tube containing cells and then incubated for 10 min at RT. The cells were immediately analyzed with BD Accuri^TM^ C6 flow cytometer (BD Biosciences, Heidelberg, Germany).
